# Genome-wide identification of *LHT* gene family in *Lonicera macranthoides* Hand.-Mazz and their responses to abiotic stresses

**DOI:** 10.3389/fgene.2025.1614541

**Published:** 2025-07-01

**Authors:** Zhaowu Li, Yue Hu, Chunzhi Liang, Lu Chen, Yao Hu, Xiaoqiu Wu, Xia Chen

**Affiliations:** ^1^ Puai Medical College, Shaoyang University, Shaoyang, China; ^2^ Department of Nephrology and Rheumatism Immunity, The First Affiliated Hospital of Hunan University of Medicine, Huaihua, China

**Keywords:** Lonicera macranthoides Hand.-mazz, AAT, LHT, genome-wide, abiotic stresses

## Abstract

Amino acid transporters (AATs) allow the transport of amino acids and play important roles in the various physiological processes and environmental responses of plants. The lysine and histidine transporter (LHT) subfamily is an important type of AAT. However, a genome-wide overview of the *LHT* gene family has not been conducted in *L. macranthoides* Hand.-mazz. In this study, 11 *LHT* genes were identified in the *Lonicera macranthoides* genome. To further understand the functions of *LmLHT* genes, the gene and protein characteristics, transmembrane helices, evolutionary relationships, chromosomal distribution, *cis*-acting elements of promoters, and expression patterns were systematically analyzed. According to the results, *LmLHT* genes were divided into two groups based on the phylogenetic analysis. Transmembrane helices of LmLHT proteins ranged from seven to 16. Gene structure and conserved motif analysis revealed that exon-intron structures and motifs were relatively conserved in the LmLHT family. *LmLHT* genes were distributed on six of the nine chromosomes and had the most collinear gene pairs with *NtLHT* genes. Additionally, phytohormones, low-temperature, drought-inducibility, defense and stress related *cis*-acting elements were enriched in the promoters of *LmLHT* genes. *LmLHT* genes showed distinct or preferential expression patterns in various tissues, signifying their potential roles in plant growth and development. We also found that some *LmLHT* genes were responsive to cold and drought stresses, indicating their roles in abiotic stress adaptation. Overall, our results provided comprehensive insight into the *LmLHT* gene family and will be useful for future functional analyses.

## Introduction

Nitrogen (N) is the major nutrient factor for plant growth and development, and plants can absorb and utilize inorganic and organic N in soil. Organic N includes amino acids, peptides and proteins (Cai and Aharoni, 2022). Approximately 40% of organic N in soil comprises peptides and proteins, and 45%–50% of soil organic N comprises amino acids (Cao et al., 2016). The concentrations of total amino acids in soil may be as high as 150 μmol/L (Weigelt et al., 2005). Amino acids and their derivatives not only participate in N fluxes both in poor and fertile soils, but also play an important role in the N cycle in plant organs (Inselsbacher and Näsholm, 2012). For plants, amino acids are essential for the activities of enzymes and proteins. Moreover, amino acids are important components of chlorophyll, polyamines, phytohormones, creatinine and nucleotides (Dong et al., 2020; Yahyaoui and Pérez-Frías, 2020). Therefore, these acids play important roles in the entire life cycle of plants, including regulating the defense response and N utilization efficiency, affecting the quality and biomass, changing the root or shoot morphology, and act as precursors of secondary metabolites (Perchlik and Tegeder, 2017; Guo et al., 2020b; Guo et al., 2021).

Generally, proteins and peptides in organic N cannot be directly absorbed by plant roots, while amino acids can be obtained by roots and transported to shoots or leaves through xylem and phloem (Näsholm et al., 2009; Tegeder and Masclaux-Daubresse, 2018). As the level of amino acids in the soil is significantly lower than that of root cells, membrane localized amino acid transporters (AATs) are necessary for the uptake of amino acids from soil (Tegeder, 2014). Currently, plant AATs are divided into three main families: the amino acid/auxin permease (AAAP) family, the amino acid polyamine and choline (APC) transporter family, and the usually multiple amino acids move in and out transporter (UMAMIT) family (Cao et al., 2024). The AAAP family contains eight subfamilies: proline transporters (ProTs), amino acid permeases (AAPs), auxin transporters (AUXs), lysine and histidine transporters (LHTs), aromatic and neutral amino-acid transporters (ANTs), γ-aminobutyric acid transporters (GATs), amino acid transporter-like proteins (ATLs), and vesicular aminergic-associated transporters (VATs) (Yang et al., 2020). The APC family contains three subfamilies: cationic amino acid transporters (CATs), L-type amino acid transporters (LATs), and polyamine H+-symporters (PHSs) (Saha and Gupta, 2022; Du et al., 2024). Among these families, AAP, LHT, and ProT are the most studied AATs in plants (Fan et al., 2023).

AtAAP1 was the first identified amino acid transporter in plants and mediates the transport of neutral and charged amino acids (Perchlik et al., 2014). Overexpression of AtAAP1 significantly increases the transport of chlorantraniliprole–alanine conjugate (Ren et al., 2019). AtAAP2 functions in the long distance transfer of amino acids, and the growth and development of leaves are enhanced in the *atapp2* mutant, which in turn increases seed yield and oil content (Elashry et al., 2013). *AtAAP3* is mainly expressed in the roots and can transport basic amino acids, such as lysine, histidine, and arginine (Okumoto et al., 2004). The root-knot nematode (RKN) infestation levels and egg mass number in the *atapp3* mutant were notably decreased, indicating that AtAAP3 may act as a positive regulator for plants against RKN invasion (Marella et al., 2012). In total, 19 *OsAAP* genes have been identified in the rice genome. OsAAP1 is located in plasma and nuclear membranes, its overexpression increases tiller numbers and fills grains, while the *osaap1* mutant presents the opposite phenotype (Ji et al., 2020). Lysine and arginine can be transported by OsAAP3, overexpression of OsAAP3 increases the accumulation of these amino acids, but reduces the number of tillers and fills grains in transgenic plants (Wei et al., 2021). OsAAP12 is located in the plasma membrane, the number of tillers is decreased in *OsAAP12* overexpression plants but increased in *osaap12* mutant lines (Jin et al., 2024).

AtLHT1 was the first identified transporter of the lysine and histidine transporter family. *AtLHT1* exhibits high expression in roots, flowers, and siliques. Overexpression of *AtLHT1* improves N efficiency and promotes plant growth, whereas disruption of *AtLHT1* affects amino acid uptake and inhibits plant growth (Hirner et al., 2006). In addition, AtLHT1 is associated with the transport of 1-aminocyclopropane-1-carboxylic acid, an important precursor of ethylene (Shin et al., 2015). *AtLHT4* has strong expression in anthers, which suggests that *AtLHT4* may be involved in anther and pollen development (Rabby et al., 2022). There are six *OsLHT* genes (*OsLHT1*-*OsLHT6*) in the rice genome, and the most studied member is *OsLHT1*. OsLHT1 is strictly located in the plasma membrane and participates in the transport of Asparagine (Guo et al., 2020b). Knockout of *OsLHT1* decreases the concentration of amino acid in xylem sap, and the translocation of amino acid in the shoot is restricted, resulting in reduced plant height, stem length, and grain yield (Guo et al., 2020a). Notably, loss of function of *OsLHT1* increases the expression of defense-responsive genes and produces more salicylic acid and jasmonic acid. Therefore, rice blast disease resistance is improved in *OsLHT1* mutants (Guo et al., 2023). OsLHT5 was localized in the cytosol and plasma membrane. The expression of *OsLHT5* was downregulated when treated with PEG and NaCl, which suggests that *OsLHT5* may play a role in abiotic stress response in rice (Fan et al., 2023). There were 23 *NtLHT* genes (*NtLHT1*-*NtLHT23*) in tobacco, and the overexpression of *NtLHT1* accelerated leaf senescence and affected leaf morphology. Moreover, the expression of *NtLHT1* was increased under abiotic stress, and the germination rate in *NtLHT1-*overexpressing plants was significantly higher than in *ntlht1* mutants, which suggests that *NtLHT1* may be involved in abiotic stress tolerance in tobacco (Xing et al., 2024). NtLHT22 was localized in the plasma membrane. The amino acid content was significantly altered in *NtLHT22-*overexpressing plants and *ntlht22* mutants, which implies that *NtLHT22* participates in amino acid homeostasis in tobacco (Li Z. et al., 2022).

*Lonicera macranthoides* Hand.-Mazz. is a species of the Caprifoliaceae family, also known as “shanyinhua” or “mountain honeysuckle” (Liu et al., 2024). It is widely planted in Southwestern China, including Hunan Province, Guizhou Province, and Chongqing City. The fresh and dried flower buds of *Lonicera macranthoides* are used as important ingredients in traditional Chinese medicine to treat and prevent fever, furuncles, inflammation, and cardiovascular diseases (Pan et al., 2021). The *LHT* gene family is important for plant growth, development, and quality. In this study, we have systematically and comprehensively identified the *LHT* gene family in *L*. *macranthoides*. The physicochemical characteristics, phylogenetic evolutions, gene architecture, chromosomal localization, gene collinearity, and *cis*-acting elements in the *LmLHT* gene family were analyzed. Additionally, the expression patterns of *LmLHT* genes in different tissues and abiotic stresses were examined using quantitative RT-PCR (qRT-PCR). Our research provides useful insights for future functional analysis of *LmLHT*.

## Materials and methods

### Plant materials

*L*. *macranthoides* Hand.-mazz plants were cultured in a greenhouse with a cycle of 16 h of light, 8 h of darkness, 22°C–24 °C. Different tissues (root, stem, leaf, white flower, and yellow flower) were collected at 6 months to analyze the expression of *LmLHT*. For cold stress treatment, four-week-old seedlings were exposed in a growth chamber at 4 °C for 5 h. For drought stress treatment, four-week-old seedlings were not watered until the leaves were completely wilted (Xie et al., 2021). The leaves of six sample plants were mixed, frozen with liquid nitrogen and then stored at −80 °C for future use.

### Identification of LmLHT gene family

The *AtLHT* genes retrieved from TAIR (http://www.arabidopsis.org) were used as a query to search in the *L*. *macranthoides* genome with an E-value cutoff ≤0.01 (Rhee et al., 2003; Yin et al., 2023). Subsequently, the Hidden Markov Model profile of the LHT domain (PF01490) was obtained from the Pfam database, and the candidate LmLHT protein sequences were confirmed on the Conserved Domain Database (CDD) of the NCBI (https://www.ncbi.nlm.nih.gov/cdd/) (Sonnhammer et al., 1997; Marchler-Bauer et al., 2015). The identified LmLHT protein sequences were renamed according to their chromosomal locations. Moreover, the basic information and chemical parameters of the LmLHT proteins were analyzed with the online tool ExPASy (http://web.expasy.org/protparam/).

### Transmembrane, protein structure and phylogenetic analysis

The transmembrane domain and protein structure of the LmLHTs were analyzed with the online tool PROTTER (http://wlab.ethz.ch/protter/start/) and PHYRE server v2.0 (http://www.sbg.bio.ic.ac.uk/phyre2/html/page.cgi?id=index), respectively. The protein sequences of 11 LmLHTs, 6 OsLHTs, 10 AtLHTs, and 23 NtLHTs were aligned using ClustalX software (version 2.1). Subsequently, a neighbor-joining (NJ) phylogenetic tree was built using MEGA X software (version 10.1.8) with 2000 bootstraps.

### Gene structure and conserved motif analysis

The coding sequence and genomic sequence of each *LmLHT* genes were submitted to the GSDS tool (http://gsds.cbi.pku.edu.cn) to analyze the gene structure. The conserved motifs of the LmLHT proteins were identified using the MEME tool (http://meme-suite.org/tools/meme).

### Chromosomal distribution and collinearity analysis

The chromosomal distribution of the LmLHT genes was mapped and annotated using the MG2C tool (http://mg2c.iask.in/mg2c_v2.0/). Collinear gene pairs between *L*. *macranthoides*, tobacco, rice, and *Arabidopsis* were investigated with the MCScanX tool (Wang et al., 2012). Subsequently, the results were drawn using Circos (Krzywinski et al., 2009).

### Promoter analysis of *LmLHT* genes

The promoter sequences about 2,000 bp upstream of the start site of the *LmLHT* genes were obtained from the *L*. *macranthoides* genome (Yin et al., 2023), and the obtained sequences were submitted to PlantCARE (http://bioinformatics.psb.ugent.be/webtools/plantcare/html/) to analyze the *cis*-regulatory elements, and the results were displayed using TBtools (Chen et al., 2020).

### Total RNA isolation and qRT-PCR

Total RNA was isolated from frozen samples with a FastPure Plant RNA Isolate Kit (Vazyme, Nanjing, China). The quality of total RNA was detected using NanoDrop One (Thermo Scientific, Waltham, MA, United States). The first strand cDNA was generated from 1 μg of total RNA with a cDNA Synthesis Kit (Vazyme, Nanjing, China). qRT-PCR was carried out using an ABI QuantStudio 3 system with SYBR Green (TIANGEN, Beijing, China), and PCR reactions were 95 °C for 5 min, followed by 40 cycles of 95 °C for 10 s and 56 °C for 30 s. The *18S rRNA* gene was adopted as the internal control, and the gene expression level was analyzed using the 2^−ΔΔCT^ method (Schmittgen and Livak, 2008; Chen et al., 2023). The primers used for qRT-PCR are provided in Supplementary Table S1.

## Results

### Genome-wide identification and basic information analysis of *LmLHT*

In this study, *Arabidopsis* LHT proteins retrieved from TAIR were used to identify candidate *LHT* genes in *L*. *macranthoides*. As a result, 11 *LHT* genes were identified in the *L*. *macranthoides* genome. To facilitate further research, we renamed the *LmLHT* genes from *LmLHT1* to *LmLHT11* in the order of their physical chromosome locations. The genomic and coding sequences of the *LmLHTs* varied from 2,151 to 4,336 bp and 1,317 to 2,355 bp, respectively. The protein length of the LmLHTs varied from 438 aa to 784 aa (Table 1). Their deduced molecular weight varied from 48.7 to 87.6 kDa, and their isoelectric point varied from 8.10 to 9.41. The major amino acids in the LmLHT proteins were leucine, glycine, and alanine, and most LmLHT proteins (except LmLHT8) were stable (instability index <40). The GRAVY values indicated that all of the LmLHT proteins were hydrophobic (GRAVY index >0). Furthermore, most of the proteins were localized in the plasma membrane (Table 2).

**TABLE 1:** Detailed information of *LmLHT* gene families.

Genes	Gene id	Chromosome no.	Start site	End site	Gene length (bp)	CDS (bp)	ORF (aa)
*LmLHT1*	*Lm3A1018T66*	3	101,852,641	101,855,300	2,659	1,317	438
*LmLHT2*	*Lm4C13T3*	4	1,357,298	1,359,827	2,529	1,398	465
*LmLHT3*	*Lm4A13T57*	4	1,375,452	1,379,014	3,562	1,581	526
*LmLHT4*	*Lm4A48T72*	4	4,875,748	4,878,985	3,237	1,545	514
*LmLHT5*	*Lm4A229T23*	4	22,931,372	22,935,516	4,144	1,581	526
*LmLHT6*	*Lm4A778T44*	5	77,785,785	77,790,357	4,572	1,548	515
*LmLHT7*	*Lm5A173T67*	5	17,380,183	17,382,762	2,624	1,378	525
*LmLHT8*	*Lm6A821T68*	6	82,127,086	82,129,237	2,151	1,398	465
*LmLHT9*	*Lm7C606G10*	7	60,684,948	60,689,284	4,336	2,355	784
*LmLHT10*	*Lm9C327T7*	9	32,780,094	32,784,397	4,303	1,359	452
*LmLHT11*	*Lm9A403T65*	9	40,305,252	40,308,513	3,261	1,380	459

**TABLE 2:** Amino acid composition and physiochemical characteristics of LmLHT proteins.

Proteins	MW	pI	Major amino acid%	Instability index	GRAVY	Localization predicted
LmLHT1	48.7	8.95	A (8.9), V (8.7), G (8.4)	35.28	0.429	chlo, plas
LmLHT2	51.5	8.98	L (8.8), G (8.6), I (8.0)	37.22	0.240	cyto, chlo, plas
LmLHT3	57.9	9.22	L (13.1), A (8.2), G (8.2)	35.78	0.518	plas, vacu
LmLHT4	56.8	9.37	L (11.9), S (9.1), F (8.0)	32.01	0.479	plas, cyto
LmLHT5	57.8	9.16	L (11.6), A (8.7), G (8.2)	37.00	0.525	plas, vacu
LmLHT6	56.7	9.41	L (12.4), S (9.9), G (8.3)	35.51	0.522	cyto, chlo, plas
LmLHT7	57.3	9.20	L (12.0), A (9.0), G (8.2)	31.14	0.554	plas, vacu
LmLHT8	52.3	8.10	L (10.8), I (8.2), S (8.0)	43.81	0.401	plas
LmLHT9	87.6	8.66	V (11.6), L (8.7), A (7.5)	29.15	0.557	plas, cyto
LmLHT10	49.9	8.87	L (12.2), G (9.1), I (8.4)	31.84	0.540	plas
LmLHT11	50.4	9.08	L (13.5), G (9.8), I (8.5)	27.75	0.648	plas

MW: molecular weight (kDa), pI: isoelectric point, GRAVY: grand average of hydropathicity, V: val, I: ile, A: ala, G: gly, S: ser, L: Leu. F: Phe. Plas: plasma membrane, Vacu: vacuoles, Cyto: cytoplasm, Chlo: chloroplast.

We also analyzed the transmembrane regions of the LmLHT proteins with the online tool PROTTER. These proteins contained 7 to 16 transmembrane domains, and most of them (91%) varied from 7 to 11 (Supplementary Figure S1). Protein structure analysis indicated that the LmLHT proteins were composed of α helices, β turns, random coils, and extended strands and had similar structures. Among these structures, α helices were the most abundant, while β turns were uncommon (Supplementary Figure S2).

### Evolutionary relationship of *LHT* genes in *Lonicera macranthoides*, tobacco, rice, and *arabidopsis*

**FIGURE 1:**
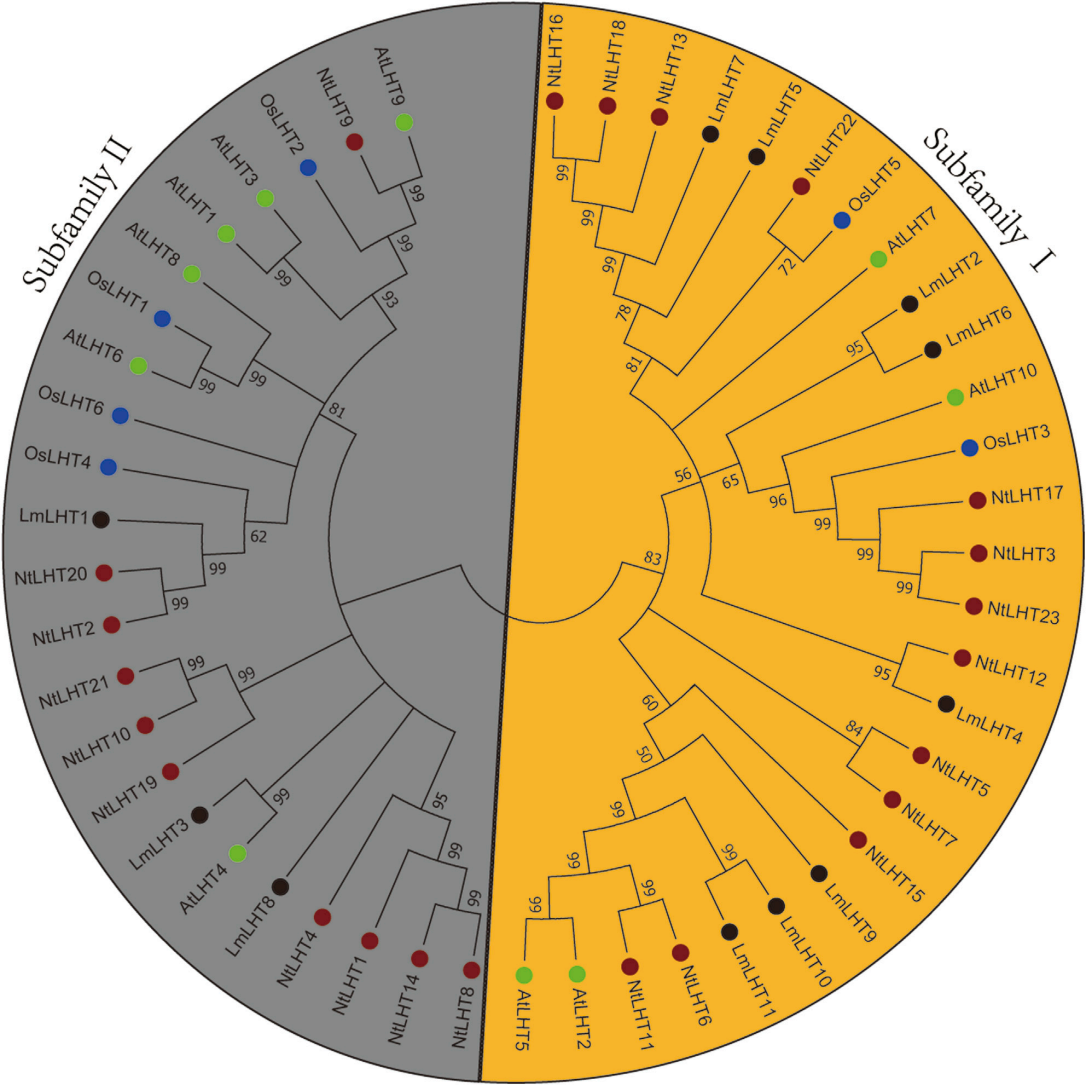
Phylogenetic analysis of LHT proteins in *Lonicera macranthoides*, tobacco, rice and *Arabidopsis*. The tree divides 11 LHT proteins from *Lonicera macranthoides*, 23 LHT proteins from tobacco, 6 LHT proteins from rice, and 10 LHT proteins from *Arabidopsis* into two subgroups. Black, red, blue, and green colors represent *Lonicera macranthoides*, tobacco, rice and *Arabidopsis*, respectively.

To explore the evolutionary relationships of the *LHT* gene family in *L*. *macranthoides*, tobacco, rice, and *Arabidopsis*, a phylogenetic tree was constructed with the neighbor-joining method for 11 LHT proteins from *L*. *macranthoides*, 23 LHT proteins from tobacco, 6 LHT proteins from rice, and 10 LHT proteins from *Arabidopsis*. As shown in Figure 1, the LHT proteins were classified into two subfamilies, which was consistent with the results of a previous study (Wang et al., 2019). Subfamily Ⅰ contained 8 LmLHT members (LmLHT2/5/6/7/8/9/10/11), 13 NtLHT members (NtLHT3/5/6/7/11/12/13/15/16/17/18/22/23), 2 OsLHT members (OsLHT3/5), and 4 AtLHT members (AtLHT2/5/7/10). Subfamily II contained 3 LmLHT members (LmLHT1/3/4), 10 NtLHT members (NtLHT1/2/4/8/9/10/14/19/20/21), 4 OsLHT members (OsLHT1/2/4/6), and 6 AtLHT members (AtLHT1/3/4/6/8/9). These results indicated that subfamily I contained more LmLHT members than subfamily II, and the members in the same subfamily may have closer evolutionary relationships.

### Gene structure and motif analysis of LmLHT family members

**FIGURE 2:**
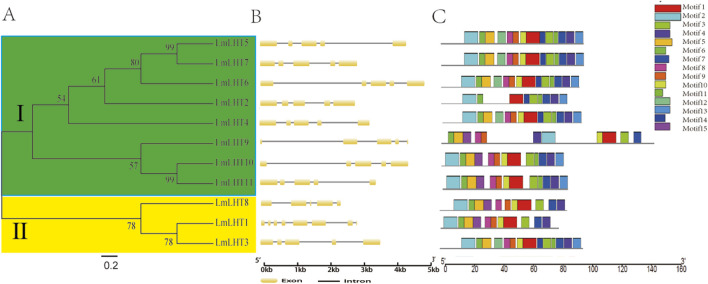
Evolutionary relationship, gene structure and motif analysis of LmLHT family members. **(A)** The phylogenetic tree was built from LmLHT proteins. The LmLHT protein sequences were divided into two subfamilies: green and yellow colors indicate subfamily Ⅰ and subfamily Ⅱ, respectively. **(B)** Exon–intron organization of *LmLHT* genes. Brown boxes represent exons, and block lines represent introns. **(C)** Conserved motifs of LmLHT proteins. The motifs are displayed in different colored boxes.

To better understand the evolution of LmLHT family members, a neighbor-joining tree was reconstructed between the *LmLHT* genes. The *LmLHT* genes were divided into two subfamilies; these results are consistent with previous research on tobacco (Li Z. et al., 2022). The gene structure and protein motifs could also provide evolutionary information. Gene structure analysis showed that all of the *LmLHT* genes had five exons and four introns, with the exception of *LmLHT1*, which had eight exons and seven introns. Furthermore, we performed a conserved motif analysis of the LmLHT proteins with MEME. It was observed that LmLHT3/4/5/6/7 contained 14 motifs, excluding motif 15, which we found to exist only in LmLHT8/9/10/11. In addition, LmLHT2 only had nine conserved motifs, which is less than the other LmLHT members, implying that some motifs were lost or degenerated during the process of evolution. It is worth noting that although LmLHT1 has the largest number of exons, it does not contain the largest number of conserved motifs. Therefore, the increased exons in LmLHT1 may act as regulatory exons rather than encoding protein motifs (Figure 2; Supplementary Figure S3). These results suggested that the conserved motifs in LmLHT family members may have undergone loss or gain during evolution.

### Chromosomal distribution and collinear analysis of *LmLHT* genes

**FIGURE 3:**
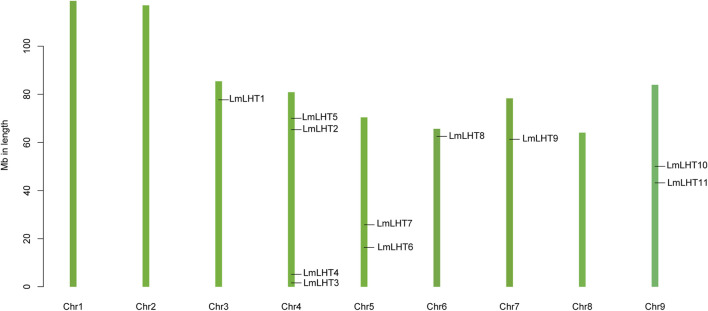
Chromosomal locations of LmLHT family members.

The localization of the *LmLHT* genes on chromosomes was identified, and it was found that they were randomly distributed on six out of nine chromosomes. Chromosome 4 contained four *LmLHT* genes, and chromosomes 5 and 9 contained two *LmLHT* genes. Only a single *LmLHT* gene was distributed on each of chromosomes 3, 6, and 7 (Figure 3).

**FIGURE 4:**
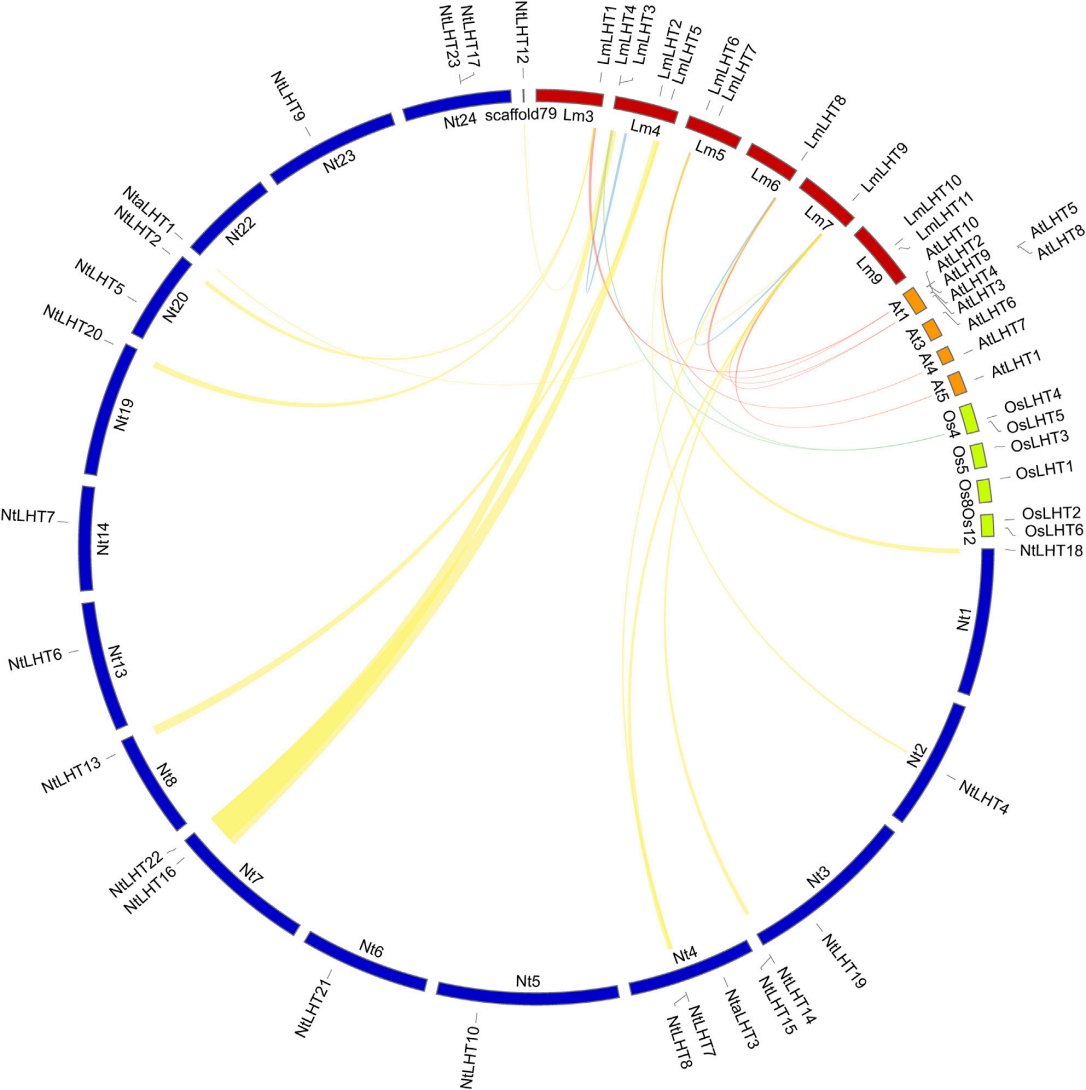
Collinearity analysis of LHT family members between *Lonicera macranthoides* (Lm), tobacco (Nt), rice (Os), and *Arabidopsis* (At). The yellow lines represent the collinear gene pairs between *Lonicera macranthoides* and tobacco, the red lines represent the collinear gene pairs between *Lonicera macranthoides* and *Arabidopsis*, the green lines represent the collinear gene pairs between *Lonicera macranthoides* and rice, and the blue lines represent the duplicated *LmLHT* genes.

Previous studies indicated that tandem, segmental, transposition, and whole genome duplication play an important role in gene duplication events (Zhang et al., 2020; Li H. et al., 2022). As shown in Figure 4, two genes (*LmLHT3* and *LmLHT4*) were tandemly duplicated on chromosome 4, and one gene pairs (*LmLHT8* and *LmLHT9*) was likely segmentally duplicated. These results suggested that tandem and segmental duplication play a crucial role in the expansion of the *LmLHT* gene family. To further investigate the evolutionary relationship between *LHT* genes, we conducted a collinearity analysis for *L. macranthoides*, tobacco, rice, and *Arabidopsis*. Collinear gene pairs between 12 of the *LmLHT* genes with *LHT* genes in tobacco were identified, followed by 6 *LmLHT* gene pairs with *Arabidopsis*, and 2 *LmLHT* gene pairs with rice. In addition, most collinear relationships between these species were one-to-one matches (Figure 4).

### *Cis*-acting element analysis of *LmLHT* genes

**FIGURE 5:**
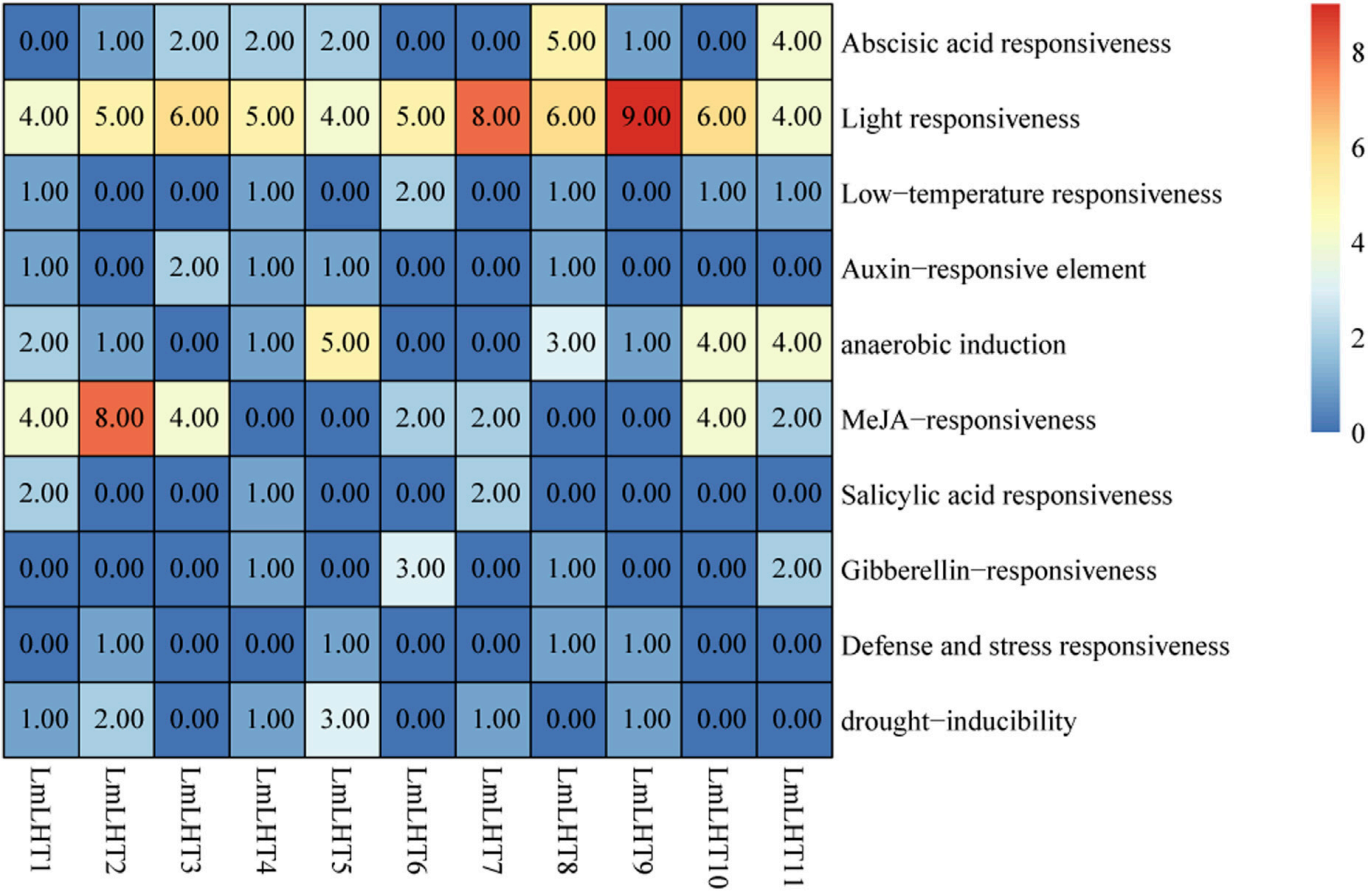
*Cis*-acting element analysis in the promoter region of *LmLHT* genes. The numbers and colors in the box represent different elements.

To better understand the regulatory mechanisms of the *LmLHT* genes, 2,000 bp upstream sequences of these genes were used for *cis*-acting element analysis. The *cis*-acting elements in *LmLHT* promoters were diverse. As shown in Figure 5, light-responsive elements were the most abundant *cis*-acting elements in *LmLHT* promoters, indicating that these elements had indispensable roles. *Cis*-acting elements involved in anaerobic induction were identified in most *LmLHT* promoters. Moreover, most of the *LmLHT* promoters contained stress (e.g., low temperature and drought inducibility)-response elements. Apart from these, *cis*-acting elements involved in hormone response, such as abscisic acid, auxin, MeJA, salicylic acid, and gibberellin, were also distributed in the promoters of the *LmLHT* genes. These results suggested that the *LmLHT* genes may exhibit different regulation features and perform different functions in various biological processes.

### Expression patterns of *LmLHT* genes in different tissues

**FIGURE 6:**
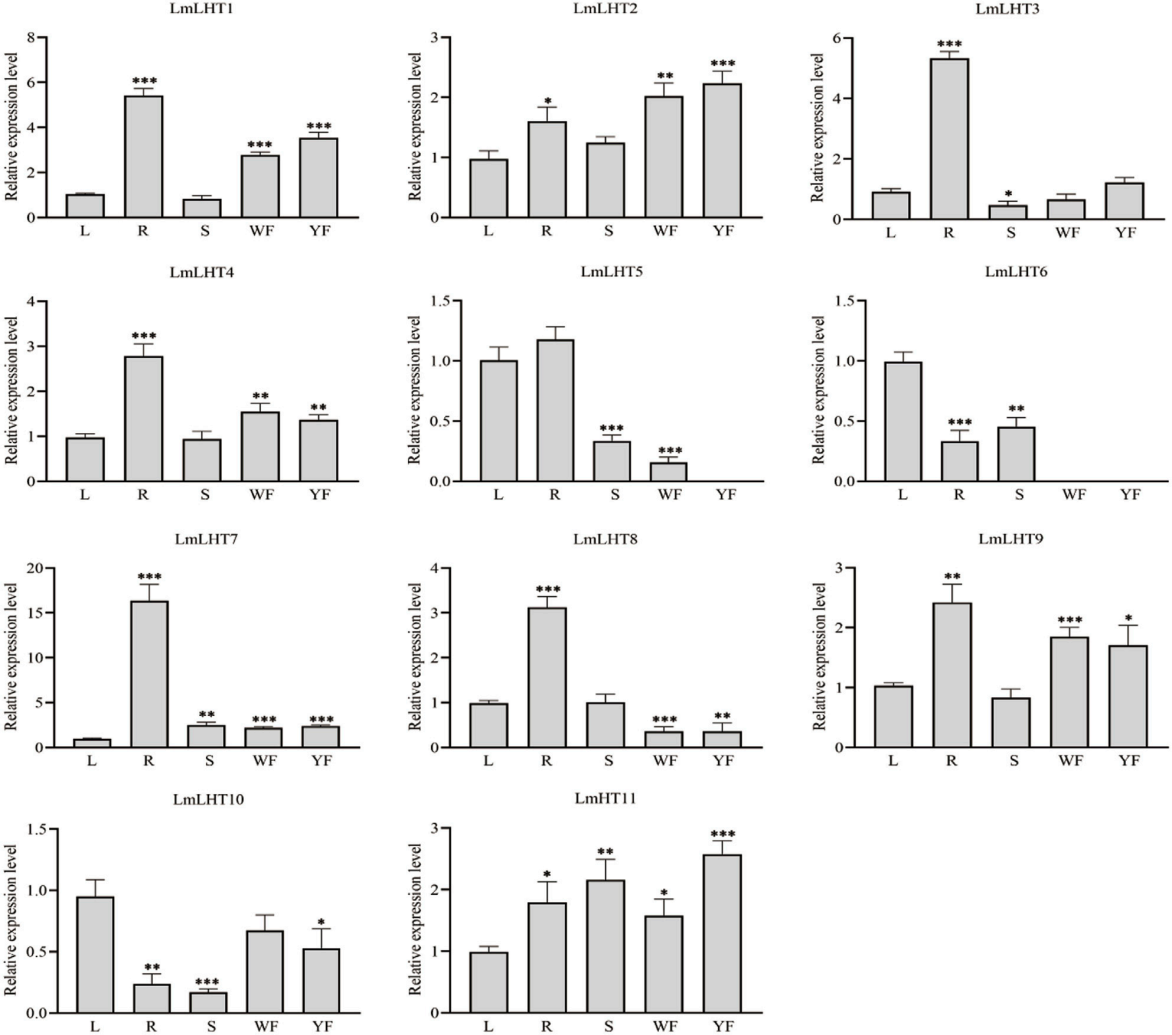
Expression levels of *LmLHT* in selected tissues. L: leaf, R: root, S: stem, WF: white flower, YF: yellow flower. Expression levels of *LmLHT* in leaves were set to one. Data are mean ± SD (n = 3).

To explore the expression patterns of the *LmLHT* genes, five tissues were selected for analysis: leaf (L), root (R), stem (S), white flower (WF), and yellow flower (YF). As shown in Figure 6, nearly half of the *LmLHT* genes, namely, *LmLHT1*, *LmLHT2*, *LmLHT4*, *LmLHT7*, and *LmLHT9*, were highly expressed in the root, white flower, and yellow flower, implying that these genes may function primarily there. In particular, the expression of *LmLHT2* in the white flower and yellow flower was significantly higher than in the other tissues, implying that it may be involved in flower development. *LmLHT11* was expressed in all the tested tissues, showing constitutive expression patterns. Furthermore, *LmLHT3* was highly expressed in the root, while *LmLHT6* and *LmLHT10* were highly expressed in the leaf. Interestingly, *LmLHT8* showed low expression in the white flower and yellow flower, *LmLHT5* showed no expression in the yellow flower, and *LmLHT6* showed no expression in the white flower and the yellow flower. In general, most of the *LmLHT* genes were preferentially expressed in vegetative tissues (root and leaf) rather than in the reproductive organ (flower).

### Expression patterns of *LmLHT* genes in response to cold and drought stresses

**FIGURE 7:**
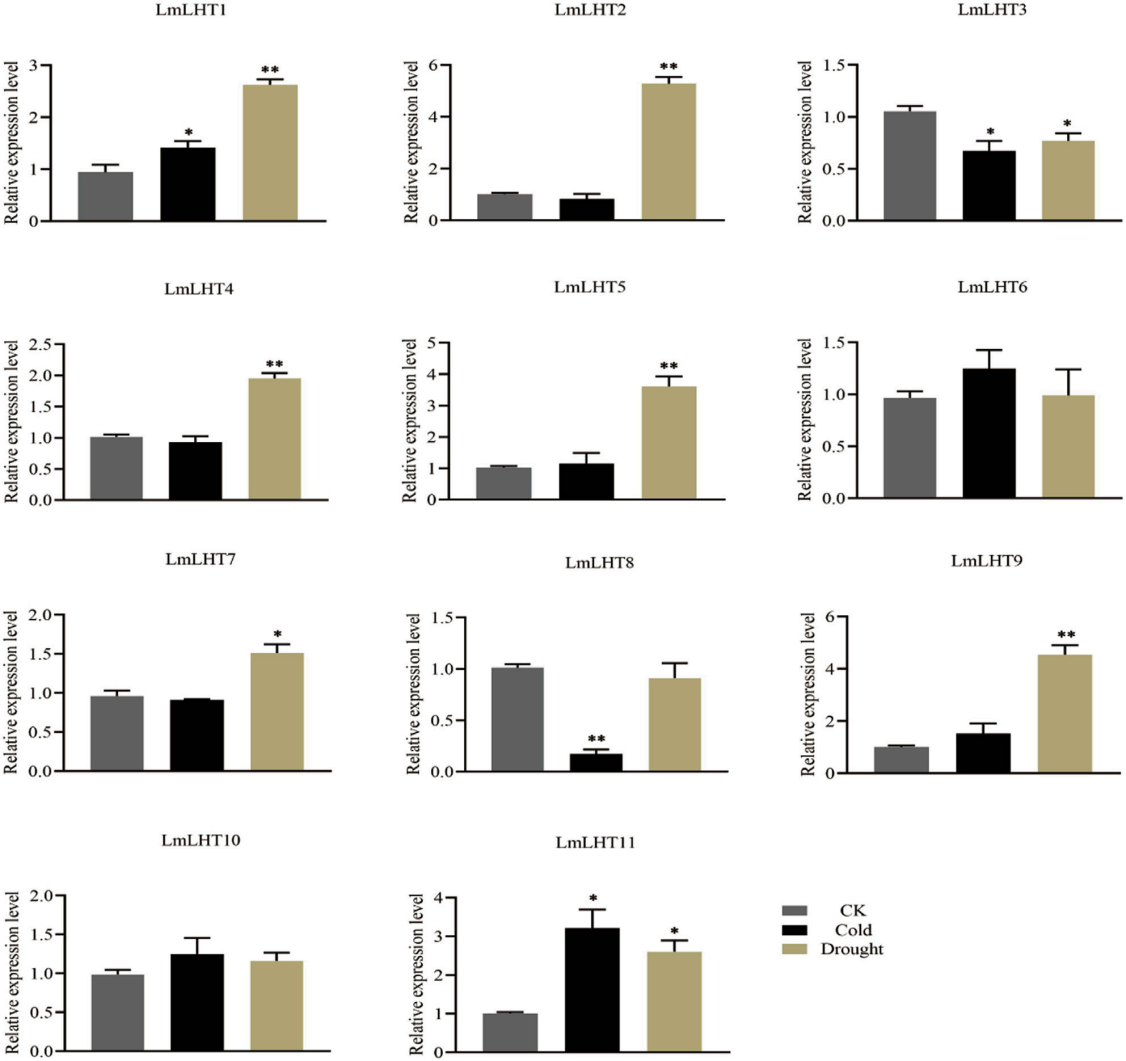
Expression levels of *LmLHT* under cold and drought stresses, seedlings under normal conditions were used as controls. Data are mean ± SD (n = 3).

Abiotic stresses often adversely affect the growth and development of plants, and previous studies have shown that *LHT* genes are responsive to various abiotic stresses (Fan et al., 2023). In this study, the expression patterns of *LmLHT* genes were investigated in response to cold and drought stresses. We found that the expression level of most of the *LmLHT* genes presented noticeable changes under cold or drought treatments (Figure 7). Under cold stress, the expression level of *LmLHT1* and *LmLHT11* was upregulated, while the expression level of *LmLHT3* and *LmLHT8* were significantly downregulated. After drought treatment, the expression level of seven genes (*LmLHT1/LmLHT2/LmLHT4*/*LmLHT5*/*LmLHT7*/*LmLHT9*/*LmLHT11*) was upregulated. By contrast, the expression of *LmLHT3* was downregulated. These results indicated that different *LmLHT* genes may have distinctive roles in response to cold and drought stresses.

## Discussion

N, including organic N and inorganic N, is a core factor in determining plant yield and quality. In most plants, N is exchanged and transported primarily in the form of amino acids. For this reason, amino acids have multiple functions in plant growth and development and are involved in various biological processes. These functions include regulating N metabolism and protein synthesis, altering root and shoot architecture, and acting as signal molecules to defend against biotic and abiotic stresses (Dong et al., 2024). AATs mediate the transport of amino acids from soil and the translocation to different tissues in plants. The AAAP superfamily is an important part of AATs, and the LHT subfamily is widely studied and functionally characterized by AAAP (Wang et al., 2019). Based on the whole genome sequencing and bioinformatics analysis, LHTs were identified in many species, including *Arabidopsis* (10 genes), rice (6 genes), tobacco (23 genes), and maize (15 genes) (Li Z. et al., 2022; Rabby et al., 2022), while the members and functions of LHTs are much less known in medicinal plants.

*L*. *macranthoides* is a famous traditional medicinal plant that is widely cultivated in Southwestern China. It has been reported that *L*. *macranthoides* plays a unique role in defending against animal and human viruses, such as the H1N1 flu virus, SARS coronavirus, and COVID-19. *L*. *macranthoides* contains a variety of biologically active compounds, including chlorogenic acid, phenolic acids, flavonoids, and organic acids (Tang et al., 2021). Increasing evidence suggests that amino acids and their derivatives possess antioxidant capacity, and the content of amino acids may provide an important contribution to the pharmacological effects of *L*. *macranthoides* (Long et al., 2024). The homeostasis and metabolism of amino acids are closely associated with AATs. However, AATs have yet to be isolated and characterized in *L*. *macranthoides*. In this study, we conducted a comprehensive identification and classification of the LHT subfamily of AATs in *L*. *macranthoides*.

We identified 11 *LHT* genes in the *L*. *macranthoides* genome, more than the number previously found in rice and *Arabidopsis* but less than that in tobacco and maize. The difference in the number of *LmLHTs* compared to other plants may be related to genome size, gene duplication, polyploidization events, and evolutionary history (Chen et al., 2025). Physiochemical properties, such as molecular weight, isoelectric point, amino acid composition, instability index, GRAVY index, and predicted subcellular localization were similar in LmLHT proteins (Table 2), which suggested that LmLHT proteins were relatively conserved during evolution. However, there are also some subtle differences. For example, the number of transmembrane helices in LmLHT proteins varied greatly (Supplementary Figure S1). Phylogenetic analysis of LHT proteins from *L*. *macranthoides*, tobacco, rice, and *Arabidopsis* indicated that these family members were categorized into two subgroups, and subgroup I possesses more LHT proteins than subgroup II (Figure 1), which is consistent with previous studies on tobacco regarding the LHT family (Li Z. et al., 2022). We also found that the distribution of OsLHTs and AtLHTs in the subgroups differs from previous studies, which may be attributed to the differences in bootstrap parameters (Wang et al., 2019). The exon–intron organization in most LmLHT family members was similar (Figure 2B), while the number of motifs in the LmLHT family members were ranged from 9 to 14 (Figure 2C). Therefore, these results imply that the number and type of motifs in LmLHT family members may be closely related with function conservation and diversification.

Gene family expansion is a common event during plant evolution, which in turn increases the gene copies for plants. Therefore, the diversity and quantity of gene families were more complex through gene duplication (Hong et al., 2024). Gene duplication events are well documented in legumes (Chen et al., 2025), grapes (Guo et al., 2025), *Arabidopsis* (Cheng et al., 2016), *etc.* Chromosome localization analysis showed that chromosome 4 contained more *LmLHT* genes (Figure 3), and this distribution pattern may be related to gene duplication. Moreover, we found that tandem and segmental duplication events occurred in the *L. macranthoides* genome. In addition, 12 gene pairs between *L. macranthoides* and tobacco, and 6 gene pairs between *L. macranthoides* and *Arabidopsis* were observed in the multi-collinearity analysis, while only 2 gene pairs were observed in *L. macranthoides* and rice (Figure 4). Thus, we speculated that the collinearity gene pairs between *L. macranthoides* and other plant species were mainly established after the differentiation of monocotyledonous and dicotyledonous plants.

*Cis*-acting elements are the non-coding DNA sequences identified in the promoter of genes, the types and number of *cis*-acting elements are closely related to gene function (Li et al., 2021). Promoter analysis of the *LmLHT* genes revealed that the most abundant *cis*-acting elements were associated with light response, suggesting that the expression of these genes might be regulated by light stimuli. Phytohormones play multiple roles in various biological processes and signal transduction, and *cis*-acting elements involved in various phytohormone responses were found in the *LmLHT* genes. At the same time, biotic and abiotic stress-related *cis*-acting elements also exist in the promoter of the *LmLHT* genes (Figure 5). Therefore, the abundant of *cis*-acting elements in the *LmLHT* genes promotor may contribute significantly to their functional diversity.

The expression patterns of genes in different plant tissues are helpful for their functional characterization. We analyzed the expression levels of the *LmLHT* genes in different parts and organs. More than half of the *LmLHT* genes showed high expression levels in the root. *LmLHT6*/*LmLHT10* are highly expressed in the leaf and *LmLHT2*/*LmLHT11* exhibited constitutive expression in the tested tissues. In addition, the expression level of *LmLHT5*/*LmLHT6/LmLHT8* in the flower is lower than in other tissues (Figure 6). These findings indicated that different *LmLHT* genes may have identical or reverse functions during growth and development. The expression of AATs in plants could be significantly influenced by abiotic stresses (Tian et al., 2020). In the present study, we found that four and eight *LmLHT* genes responded to cold and drought stresses, respectively. *Cis*-acting elements related to cold stress response were not detected in the promoters of *LmLHT3/LmLHT9*, and a drought-inducibility element was not identified in the promoter of *LmLHT11*. However, the expression of *LmLHT9* and *LmLHT11* was increased under cold and drought treatments. In addition, the expression of *LmLHT3* was decreased with cold and drought stresses (Figure 7). These results indicate that the expression of *LmLHT3/LmLHT9/LmLHT11* under cold or drought stresses may not been directly related to the *cis*-acting elements on their promoters. Instead, it may be indirectly influenced by transcription factors or epigenetic regulation. Previous studies indicated that overexpression of the light-responsive transcript factor SlBBX20 increased the expression of low-temperature responsive genes in tomato (Ma et al., 2025). The bHLH transcript factor MYC2 is a core regulator of the JA signal pathway, which participates in repeat dehydration stress in *Arabidopsis* (Liu et al., 2016). The exogenous application of ABA altered DNA methylation and stress-related gene expression in grape, thereby affecting fruit ripening (Li et al., 2024). Therefore, the complex expression patterns of the *LmLHT* genes indicate that *L. macranthoides* might have developed specialized regulatory mechanisms to adapt to environmental stresses.

## Conclusion

In summary, a systematic study was performed to identify and characterize the *LHT* family genes in *L*. *macranthoides*. The *LmLHT* genes were studied in terms of their physicochemical characteristics, evolutionary relationships, gene structure, conserved motif, chromosomal locations, collinearity, and *cis*-acting elements, which provide insights into the evolutionary history of this family in *L*. *macranthoides*. The expression patterns of *LmLHT* genes in different tissues and under cold and drought stresses were complex, suggesting that *LmLHT* genes play important roles in various biological processes. Overall, this study not only provides useful information for a comprehensive understanding of *LmLHT* genes, but also lays a solid foundation for the further application of these genes.

## Data Availability

The original contributions presented in the study are included in the article/Supplementary Material, further inquiries can be directed to the corresponding author.
