# Supplementary material for: Genome-wide identification of LHT gene family in Lonicera macranthoides Hand.-Mazz and their responses to abiotic stresses
Source: Front Genet. 2025 Jul 1;16:1614541. doi: 10.3389/fgene.2025.1614541 (PMC12259443; doi:10.3389/fgene.2025.1614541)
Supplement: Supplementary file 1 [file Table1.docx]

**Table S1.** Primers used in this study.

| List of qRT-PCR primers used in this study | |
| --- | --- |
| primer name | sequence(5'-3') |
| LmLHT1-F | AGCCCTCGAAAGTACCAATG |
| LmLHT1-R | ACGCCCAGTACCCAATAAAAG |
| LmLHT2-F | ACTCTTTGGTGCTACTTTCGG |
| LmLHT2-R | AGCTTCGATGTTGAACCCC |
| LmLHT3-F | TGGCTAAGAACAGGGTTTCG |
| LmLHT3-R | CAAGGGTATGCTAAGGTGATAGG |
| LmLHT4-F | GCCGATGAAGAGGGTGTTAG |
| LmLHT4-R | GGCGTTGAGGTTGTGAAAAG |
| LmLHT5-F | GTTCCCAATCTTATACCTCTCGG |
| LmLHT5-R | ACCAAGAACCACTCAACCG |
| LmLHT6-F | ATGGAAACCTTATGCCCTCG |
| LmLHT6-R | CCTCTAGAAATGTCGTGGCTG |
| LmLHT7-F | TTGAACTCTATAGCAGGCGTG |
| LmLHT7-R | GAAACCCTAGCAATCTCCGTAC |
| LmLHT8-F | GTCGGTACAAGCATCGTCTAC |
| LmLHT8-R | GCAAAGTGAACAGAGGCAAAG |
| LmLHT9-F | TCTCACCTTCCCAACTTCAAC |
| LmLHT9-R | TGTGGTCTTCGCTTTGTATCC |
| LmLHT10-F | AGCCCTTTTACTTCCTGTCG |
| LmLHT10-R | TGATTCGTGGAGATGGACAAG |
| LmLHT11-F | GCATTTGTGGCTCTTGGATG |
| LmLHT11-R | TGCGATTGAAAGGTGGAGG |
| 18SrRNA-F | AGGCGCGCAAATTACCCAATCC |
| 18SrRNA-R | GCCCTCCAATTGTTCCTCGTTAAG |
