# Supplementary figures and images for: Genome-wide identification of LHT gene family in Lonicera macranthoides Hand.-Mazz and their responses to abiotic stresses
Source: Front Genet. 2025 Jul 1;16:1614541. doi: 10.3389/fgene.2025.1614541 (PMC12259443; doi:10.3389/fgene.2025.1614541)

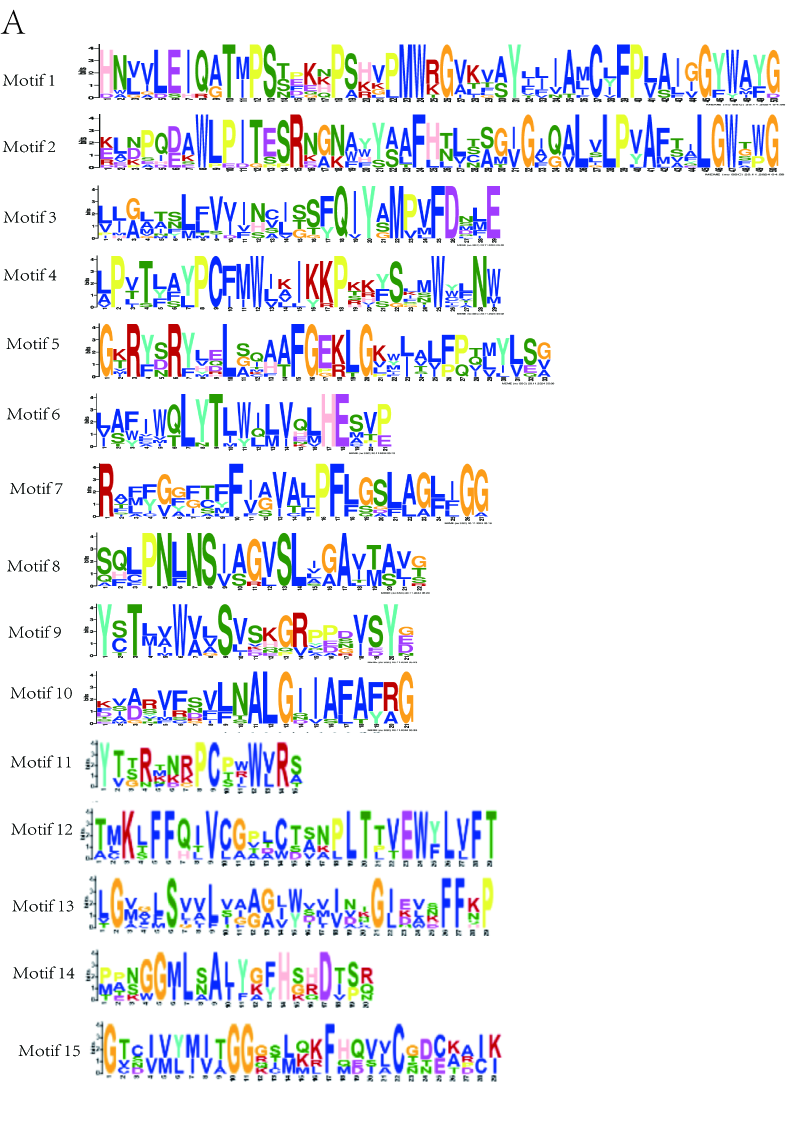

Supplement: Supplementary file 2 [file Image3.tif]

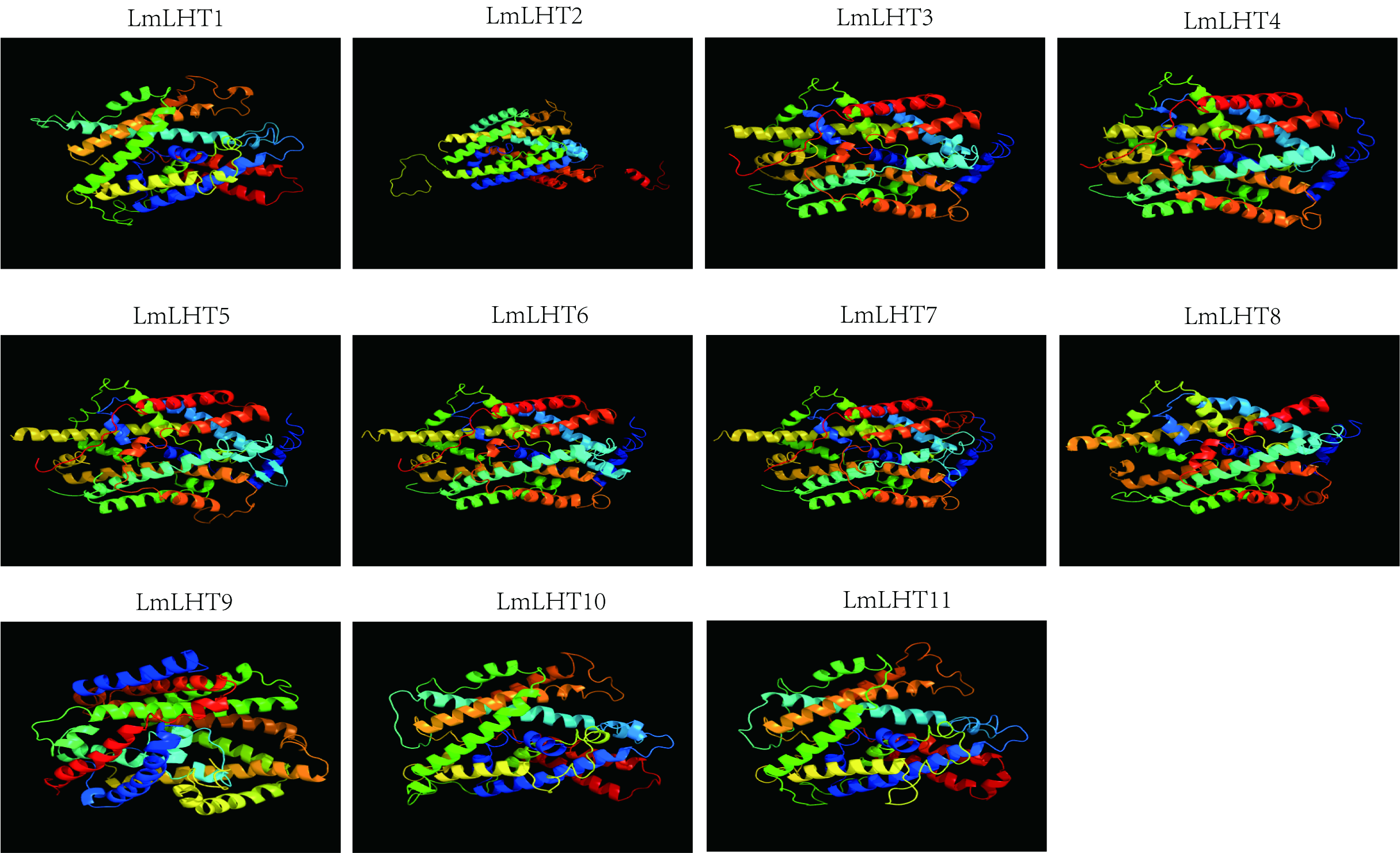

Supplement: Supplementary file 3 [file Image2.tif]

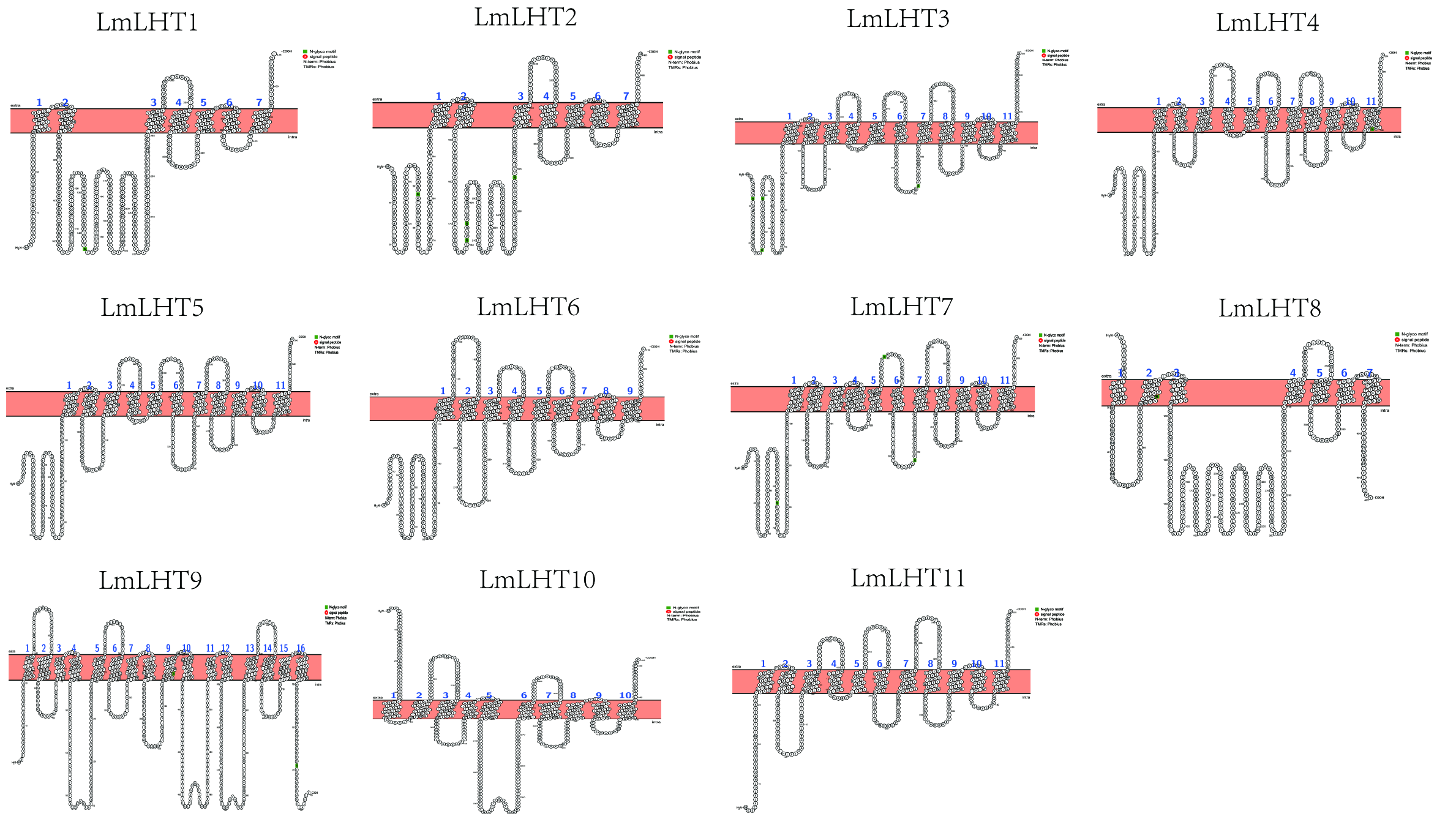

Supplement: Supplementary file 4 [file Image1.tif]
